# Multiple Osteomas on the Skull: A Case Report and Literature Review

**DOI:** 10.7759/cureus.113627

**Published:** 2026-07-29

**Authors:** Ramachandra Reddy Gowda Venkatesha, Karthik Rajaram Mohan, Mathi Vadhana Ramasamy Shanmugam Vijayan, Reethika Rathan, Ignatious Jeba Mary R

**Affiliations:** 1 Oral Medicine and Radiology, Vinayaka Mission's Sankarachariyar Dental College, Vinayaka Mission's Research Foundation (DU), Salem, IND

**Keywords:** benign bone tumor, frontal bone, osteoma, panoramic radiography, skull

## Abstract

An osteoma is a noncancerous growth that can develop from dense or spongy bone, typically located in the facial area or the skull. It is widely considered that the occurrence of osteoma is not accurately documented, as the majority of cases do not show any symptoms. This article enlightens us on the types of osteomas, etiological hypotheses, clinical features, and treatment.

## Introduction

Osteomas can form from membranous bones of the skull and face [1]. The cause of slow-growing osteoma is obscure, but the tumor may arise from the cartilage or embryonal periosteum [1]. This lesion may be solitary or multiple, occurring on a single bone or numerous bones [1]. Osteomas originate from the periosteum and may occur externally, within the paranasal sinus, or intracranially within the dura mater [2].

The classification of osteomas as either benign neoplasms or hamartomas is ambiguous. This lesion can be multiple or solitary lumps and develop on a single or multiple bones. Osteomas arise from the periosteum and can be found externally, in the paranasal sinus, or intracranially within the brain parenchyma or falx near the dura mater. Osteomas are more prevalent in the frontal and ethmoidal sinuses compared to the maxillary sinus. Osteomas are classified into three forms based on their structure: (1) ivory osteoma, which is formed of compact bone; (2) cancellous osteoma, which is composed of cancellous bone; and (3) a combination of compact and cancellous bone [1,2].

Osteomas occur at any age but most frequently are found in individuals older than 40. The early symptom of a developing osteoma is the asymmetry caused by bony, hard swelling. The swelling is usually painless unless it is associated with function. The osteomas are generally attached to the cortical plate along a broad base. The mucosa covering the tumor is normal in color and freely movable. Cortical-type osteomas develop more often in men, whereas women have the highest incidence of cancellous type. Although most osteomas are small, some can grow to large or giant-sized lesions enough to cause severe damage, especially in the frontoethmoidal region [3]. Osteomas are more common in the frontal and ethmoidal sinus than in the maxillary sinus [3]. 

Men are more prone to developing cortical-type osteomas. The highest occurrence of cancellous type is among females. While most osteomas are minor, there are instances where they can develop into big or giant-sized lesions that can potentially cause significant harm, particularly in the frontoethmoidal region [3]. Osteomas can occur in the hard palate in the maxilla [4]. Osteomas occurring in the sphenoid bone can lead to unilateral headache [5]. This case report aims to describe the clinical features of asymptomatic multiple osteomas in the frontal bone.

## Case presentation

**Figure 1:**
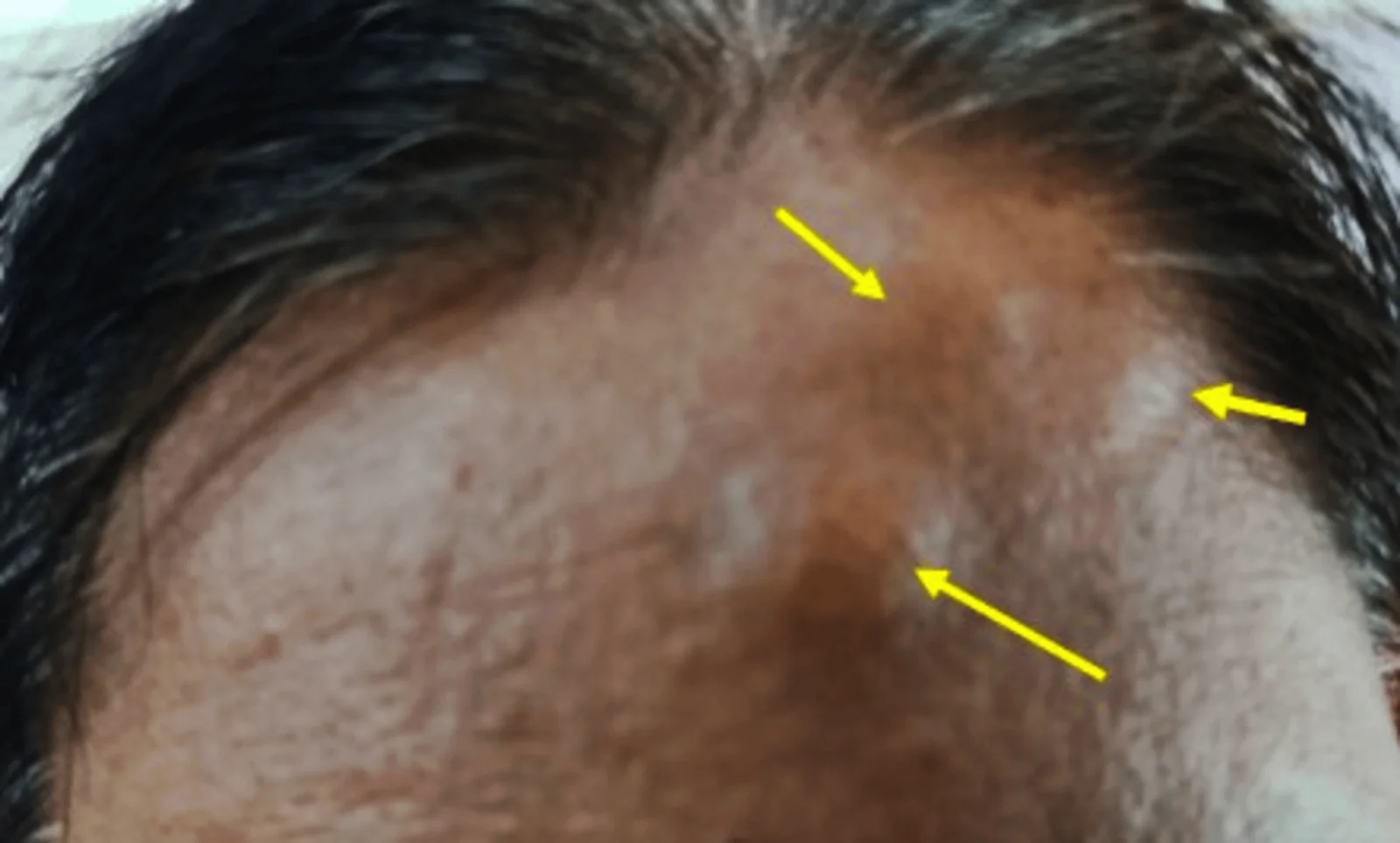
Clinical examination revealed three discrete lumps on the left side of the forehead (yellow arrows)

A 62-year-old South Indian Dravidian female reported to our Department of Oral Medicine and Radiology for a routine dental checkup. Her medical history revealed she was not diabetic, hypertensive, or associated with any other systemic illness. She also did not give any previous history of hospitalization for major illness. An extraoral examination revealed swelling on her forehead region. History revealed that the swellings on her forehead were present for a period of seven years and were completely asymptomatic. On inspection, three discrete swellings are seen measuring about 1.5 cm x 2 cm, 1.5 x 1 cm, and 0.5 x 1 cm on the forehead (Figure 1).

**Figure 2:**
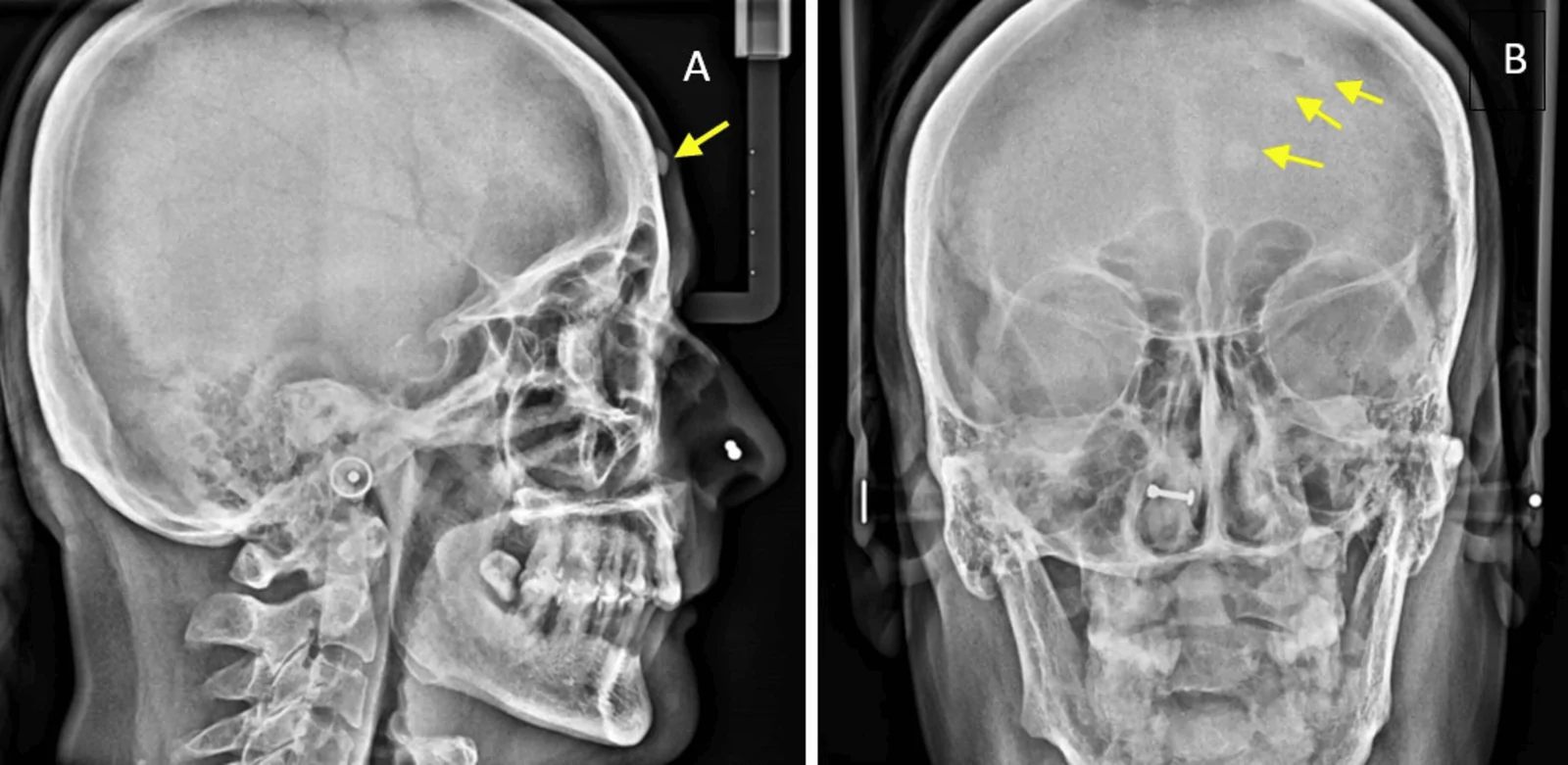
(A) Lateral cephalometric radiograph revealed a radiopaque mass attached to the outer cortical plate (yellow arrow). (B) Anteroposterior skull view revealed three discrete radiopacities on the frontal skull bone of varying diameters (yellow arrows) Patient informed consent was obtained for the publication of her radiographic images. The declaration of Helsinki is followed in this case report

On palpation it was nontender, bony hard in consistency, and sessile with a broad base. Intraoral examination revealed impacted left maxillary third molar, generalized periodontitis. The lateral cephalometric radiograph revealed the presence of a well-defined radiopaque mass with a broad base attached to the outer cortical border of the frontal bone, and the anteroposterior skull view revealed three discrete radiopacities of varying diameters on her left side of the frontal bone (Figure 2A,2B).

**Figure 3:**
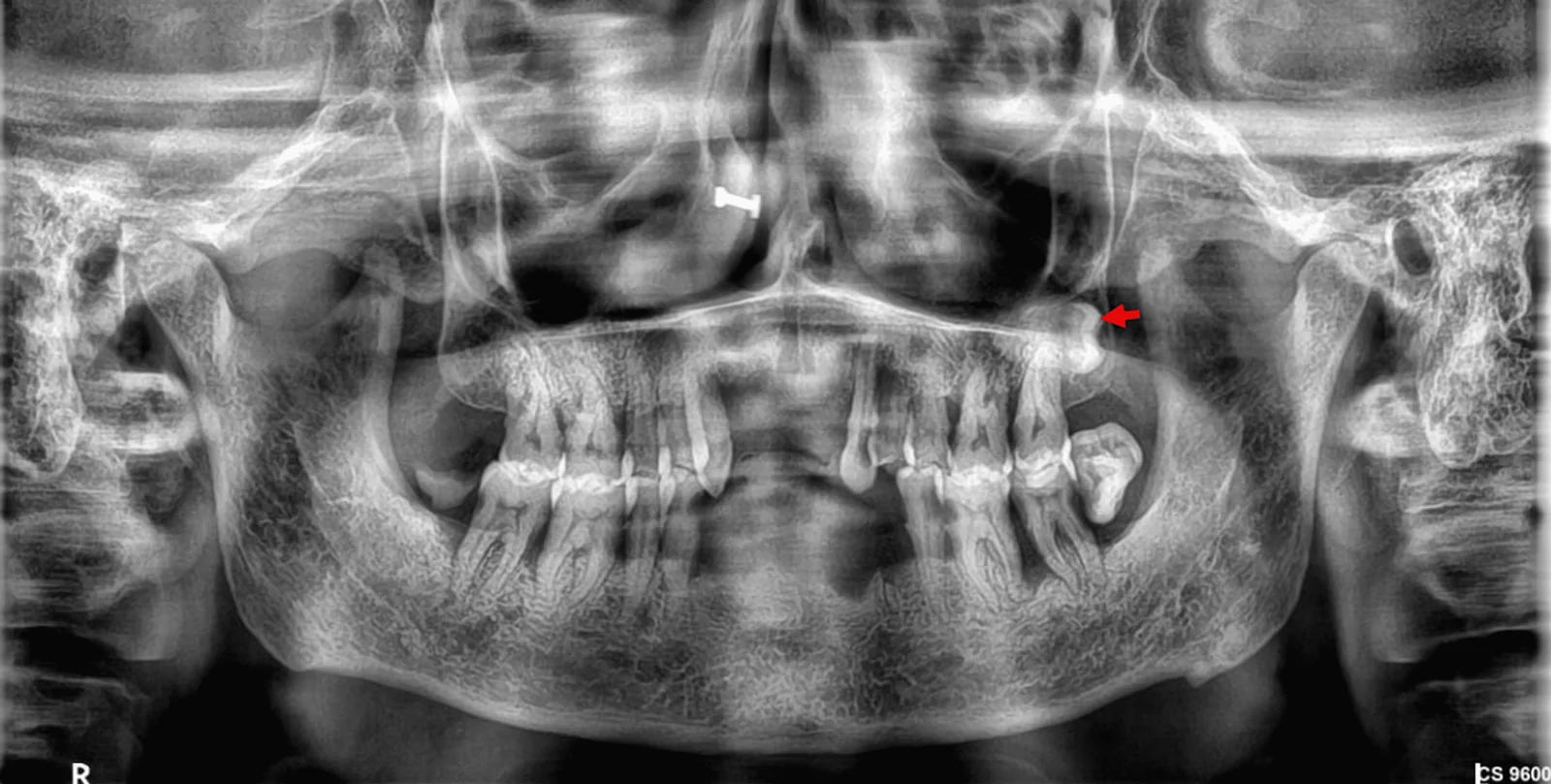
Digital orthopantomography revealed generalized alveolar bone loss and horizontal impacted left maxillary third molar (red arrow)

Panoramic radiography revealed impacted left maxillary third molar, generalized alveolar bone loss, proximal caries in her left maxillary canine, mandibular second premolar, mandibular first molar, floating teeth appearance concerning her left mandibular third molar, root stump in mandibular third molar, missing maxillary and mandibular right and left central and lateral incisors, mandibular canine, and longer roots (Figure 3).

The patient was recalled and followed up after a month; there was no increase in the size of the lump. The patient was completely asymptomatic. Hence, no surgical intervention is required in this case.

## Discussion

An osteoma is a noncancerous growth that can develop from either dense or spongy bone and is typically located in the face or skull. It is widely considered that the occurrence of osteoma is not accurately reported because most cases do not show any symptoms [2]. It is imperative to take into account the radiographic modality when diagnosing patients with osteoma [2]. The classification of osteomas based on their composition and anatomical location is enumerated in Table 1.

**Table 1:** Classification of osteomas

Based on the type of bone	Clinical features
Compact osteomas	It is composed of mature, compact lamellar bone
Spongy osteomas	It is composed of marrow and trabecular bone
Based on the location	
Central osteomas (intraosseous osteomas)	Intraosseous osteomas are located within the bone
Peripheral osteomas (extraosseous osteomas)	Extraosseous osteomas located in the gingiva
Extraskeletal osteomas (soft tissue osteomas)	It occurs in soft tissue, usually in the oral cavity and skin
Internal auditory canal (IAC) osteomas	Osteomas in the internal auditory canal result in tinnitus, vertigo, ear pain, or hearing loss
Cranial osteomas	Osteomas in the cranial bone of the skull, are often asymptomatic, no pain, and unaesthetic
Intraparenchymal	Osteomas within the brain parenchyma
Dural osteomas (brain stones)	Osteomas located near dural falx along the superior sagittal sinus and do not have any communication with bone
Skull base	Osteomas occur in the base of the skull near the clivus
Skull vault osteomas	Osteomas occur in the vault of the skull
Enostotic osteomas	Osteomas arise from the inner table of the skull
Exostotic osteomas	Osteomas arise from the outer table of the skull
Retromastoid osteomas (temporal bone osteomas)	Osteomas occur within the temporal bone
Paranasal sinus osteomas	Osteomas occur within the paranasal sinus frontal, ethmoidal, and maxillary sinus
Frontal sinus osteomas	Osteomas within the frontal sinus
Ethmoid sinus osteomas	Osteomas within the ethmoidal sinus. Ethmoidal sinus osteomas progress slowly, can penetrate the skull base and cause recurrent sinusitis
Sphenoid sinus osteomas	Osteomas in the sphenoidal sinus. Sphenoidal sinus osteomas cause headache, deformity of orbit, pneumocephalus, rhinorrhea, meningitis, and abscess formation
Osteoid osteomas	Osteoid osteomas are benign, slow-growing tumors that usually occur in the long bones of lower extremities, such as the femur
Intracortical osteoid osteomas	Occur intracortical within long bones
Subperiosteal osteoid osteomas	Occur beneath the periosteal layer in long bones, hands, neck of the talus, feet, and femoral neck (medial aspect)
Medullary or endosteal osteoid osteomas	Occur within the medullary cavity in long bones

The formation of osteomas is attributed to various etiological hypotheses. The reactive theory, which posits that peripheral osteoma is caused by a combination of trauma and muscle activity, is widely considered the most accurate explanation for its pathophysiology [4]. Trauma can result in bleeding or swelling beneath the periosteum, and the pulling of muscles can cause the periosteum to rise, triggering a bone-forming response [4]. The initial trauma may be insignificant and easily overlooked by the patient [4]. The persistent muscular tension in the region could sustain the response, leading to the development of the tumor [4].

There are three distinct categories: the central osteoma arises from the endosteum; the peripheral type comes from the outermost layer of bone, the periosteum; and the extraskeletal form grows in soft tissues, usually in the oral cavity [3,4].

Osteomas are discovered coincidentally during imaging studies conducted for other neurological problems [4]. Orbital cellulitis occurs due to a sizeable sino-orbital osteoma [4]. Osteoma appears as a pedunculated bony mass in the hard palate [4]. Exophthalmos and one-sided headache are symptoms resulting from an osteoma located in the larger greater wing of the sphenoid bone [5]. Osteomas were most frequently seen in the external auditory canal (66%), followed by the mastoid bone (21%) and least in the middle ear cavity (13%) [6]. Giant-sized osteomas are those arising from the outer table of cranial bone of size greater than 3 cm in diameter [7]. Retromastoid osteomas can cause a sensation of pressure due to the protrusion of such osteomas during sleeping on the site of location [7]. The occurrence of the frontal bone and paranasal sinus involvement is more common than the involvement of the jaw bones [8]. Some structures were located between the dura and arachnoid mater, causing the nearby brain tissues to compress and leaving impressions on them. Additionally, there were structures located outside the dura mater [8]. Osteomas can occur in the mandible after tooth extraction [9]. Nilesh et al. reported a case of central compact osteoma, which occurred in the mandibular condyle [10]. Temporal bone osteomas are infrequently observed [9]. Internal auditory canal osteoma is an uncommon occurrence [10]. Gardner's syndrome is associated with several multiple osteomas of the skull [11]. Adarsh and Chakravarthy stated that surgical excision of osteomas is considered only for cosmetic correction and in cases of compression of adjacent vital structures [12]. When there are many abnormalities in the cranium, one should evaluate the possibility of an osteoma [13]. Cao et al. reported a case of trigeminal neuralgia caused by a petrous osteoma located at the apex of the petrous part of the temporal bone and excised surgically by a transpetrosal approach [14]. Abdulla et al. reported a case of intractable trigeminal neuralgia-like facial pain caused by an osteoma in the clivus [15].

The therapy of osteoma is determined by the patient's symptoms, with surgical intervention being recommended for symptomatic patients and regular monitoring for those with asymptomatic osteomas. Orbitotomy with craniotomy is indicated for large orbitocranial osteomas [16]. Craniectomy is the preferred surgical treatment for treating large skull osteomas [17]. The utilization of three-dimensional printing technology enabled the creation of a Patient-Specific Instrument Guide (PSIG), which effectively assisted the surgeon in extracting the skull osteoma while preserving the skull's aesthetic appearance [17]. The surgical operation procedure to remove an osteoma from the skull base involving the frontal sinus and associated pneumocephalus is called a transfrontal sinus craniotomy with pneumocephalus decompression [18]. An uninaral endoscopic endonasal approach is indicated for surgical resection of osteoid osteoma involving the anterior skull base with complaints of nasal congestion, frequent watery discharge from the nose, and occasional headache [18]. Reynard et al. reported ipsilateral vestibulocochlear compression syndrome and nervus intermedius resulting in recurrent typewriter like tinnitus, dizziness, paroxysmal vertigo, hearing loss in a 75-year-old women patient caused by bilateral internal acoustic osteomas [19]. Yang et al. documented a case where a skull osteoma relapsed following hydroxyapatite cement cranioplasty [20].

Osteomas typically have a positive, favorable treatment response, and surgery is the definitive treatment for osteomas with symptoms. Asymptomatic osteomas can be managed through observation, with regular monitoring to assure their stability, and hence, no treatment is indicated in our cases.

## Conclusions

Osteomas on the skull are benign bone growths usually asymptomatic. Osteomas are discovered incidentally during clinical examination. The symptoms of osteomas depend generally on their anatomical location in the human body. Osteomas have a favorable prognosis. Osteoma treatment is often unnecessary unless symptoms arise, in which case surgical removal is effective. Regular monitoring through radiographic examination is essential to evaluate any changes in size or symptoms associated with osteomas.

## References

[1] Putro YA, Magetsari R, Taroeno-Hariadi KW, Dwianingsih EK, Pribadi AW, Sukotjo KK (2023). Classic and rare manifestations of multiple osteoma: a case report. Int J Surg Case Rep.

[2] Arslan HH, Gökgöz MC, Cebeci S, Taşlı H (2016). Treatment approaches to temporal bone osteomas (Article in Turkish). Kulak Burun Bogaz Ihtis Derg.

[3] Bagheri A, Feizi M, Jafari R, Kanavi MR, Raad N (2021). Orbital cellulitis secondary to giant sino-orbital osteoma: a case report. Cancer Rep (Hoboken).

[4] Saxena S, Ashwin VG, Rajinikanth D (2019). A sizeable solitary pedunculated peripheral osteoma of the hard palate: a case report. Indian J Otolaryngol Head Neck Surg.

[5] Tawashi Y, Tawashi K, Beski T, Alhakeem K (2023). Exophthalmos and hemiheadache caused by osteoma in the greater wing of sphenoid bone: an extremely rare case report. Ann Med Surg (Lond).

[6] Soliva Martínez D, Belda González I, Fernández Iglesias P (2015). Osteoma of the internal auditory canal. Acta Otorrinolaringol Esp.

[7] Tan EW, Barco JB, Rehman MU, Tan CC (2020). Retromastoid osteoma-a rare case report. J Surg Case Rep.

[8] Kar A, Gaikwad M, Patnaik M (2021). A rare occurrence of multiple intracranial osteomas in the cranial cavity of a cadaver with a short review on subdural osteoma. Cureus.

[9] Wolf-Grotto I, Nogueira LM, Milani B, Marchiori EC (2022). Management of giant osteoma in the mandible associated with minor trauma: a case report. J Med Case Rep.

[10] Nilesh K, Vande A, Reddy S (2020). Central compact osteoma of mandibular condyle. BMJ Case Rep.

[11] Dinakaran N, Balakumar V, Saravanam PK (2022). Osteomas of temporal bone: a rare presentation. BMJ Case Rep.

[12] Adarsh M, Chakravarthy V (2021). Multiple giant skull osteomas associated with Gardner's syndrome. Neurol India.

[13] Tempaku A (2017). Multiple skull osteomas in a 24-year-old woman. J Gen Fam Med.

[14] Cao C, Li M, Wu M, Jiang X (2023). Trigeminal neuralgia secondary to osteoma and vascular compression: illustrative case. J Neurosurg Case Lessons.

[15] Abdulla E, Das K, Ravindra J, Shah T, George S (2022). Intractable trigeminal neuralgia secondary to osteoma of the clivus: a case report and literature review. J Neurosci Rural Pract.

[16] Yudoyono F, Sidabutar R, Dahlan RH, Gill AS, Ompusunggu SE, Arifin MZ (2017). Surgical management of giant skull osteomas. Asian J Neurosurg.

[17] Wang TH, Ma H, Huang LY (2020). Printing a patient-specific instrument guide for skull osteoma management. J Chin Med Assoc.

[18] Candy NG, Wu KC, Finger G, VanKoevering K, Prevedello DM (2024). Management of frontoethmoidal osteoma causing pneumocephalus and cerebrospinal fluid leakage with minimally invasive techniques: illustrative cases. J Neurosurg Case Lessons.

[19] Reynard P, Ionescu E, Karkas A, Ltaeif-Boudrigua A, Thai-Van H (2020). Unilateral cochleovestibular nerve compression syndrome in a patient with bilateral IAC osteoma. Eur Ann Otorhinolaryngol Head Neck Dis.

[20] Yang X, Liu Y, Zhang Y (2023). Relapse of skull osteoma after hydroxyapatite cement cranioplasty: case report. Front Oncol.

